# C4b-Binding Protein Is Present in Affected Areas of Myocardial Infarction during the Acute Inflammatory Phase and Covers a Larger Area than C3

**DOI:** 10.1371/journal.pone.0002886

**Published:** 2008-08-06

**Authors:** Leendert A. Trouw, Marcin Okroj, Koba Kupreishvili, Göran Landberg, Bengt Johansson, Hans W. M. Niessen, Anna M. Blom

**Affiliations:** 1 Department of Laboratory Medicine, Medical Protein Chemistry, Lund University, Malmö, Sweden; 2 Institute for Cardiovascular Research, VU University Medical Center, Amsterdam, The Netherlands; 3 Department of Pathology, Lund University, Malmö, Sweden; 4 Department of Cardiology, Umeå University, Norrlands University Hospital, Umeå, Sweden; 5 Department of Pathology and Cardiac Surgery, VU University Medial Center, Amsterdam, The Netherlands; University of Sheffield, United Kingdom

## Abstract

**Background:**

During myocardial infarction reduced blood flow in the heart muscle results in cell death. These dying/dead cells have been reported to bind several plasma proteins such as IgM and C-reactive protein (CRP). In the present study we investigated whether fluid-phase complement inhibitor C4b-binding protein (C4BP) would also bind to the infarcted heart tissue.

**Methods and Findings:**

Initial studies using immunohistochemistry on tissue arrays for several cardiovascular disorders indicated that C4BP can be found in heart tissue in several cardiac diseases but that it is most abundantly found in acute myocardial infarction (AMI). This condition was studied in more detail by analyzing the time window and extent of C4BP positivity. The binding of C4BP correlates to the same locations as C3b, a marker known to correlate to the patterns of IgM and CRP staining. Based on criteria that describe the time after infarction we were able to pinpoint that C4BP binding is a relatively early marker of tissue damage in myocardial infarction with a peak of binding between 12 hours and 5 days subsequent to AMI, the phase in which infiltration of neutrophilic granulocytes in the heart is the most extensive.

**Conclusions:**

C4BP, an important fluid-phase inhibitor of the classical and lectin pathway of complement activation binds to jeopardized cardiomyocytes early after AMI and co-localizes to other well known markers such as C3b.

## Introduction

During the course of several cardiovascular events cell death occurs [1]. The presence of dead cells not only impairs the function of tissues but also forms a rich source of inflammatory mediators and autoantigens [2]–[4]. Acute myocardial infarction (AMI) is one of the major causes of mortality and morbidity in the western world. Arrhythmia, cardiac rupture and acute heart failure comprise the main events leading to mortality whereas chronic heart failure is the leading cause for morbidity. If the myocardium is deprived of perfusion for several hours, prolonged ischemia results in permanent loss of function through cell death by apoptosis [5], [6]. In case reperfusion is achieved within this time frame than the extent of cell death is limited [7]. However, reperfusion as such is not just beneficial but may also have adverse effects. Cells suffer from this reperfusion, which may lead to irreversible injury, thought to occur via apoptosis and inflammation related necrosis [8]. Complicated interactions, between ischemic or dead cardiomyocytes and inflammatory cells, cytokines, acute phase proteins and complement components result in the pathologies observed in AMI [9]. The involvement of the complement system in AMI has been concluded from both animal models and human studies where the affected areas in the heart stained positive for several complement components, including C1q, mannose-binding lectin, C3 and C5b-9. Based on these and other observations it was concluded that all three pathways of complement activation participate in the process. Although the complement system often contributes to the tissue damage it is not easy to discriminate this from the beneficial effects of complement in terms of waste disposal.

C-reactive protein (CRP) is an acute phase protein in humans and it was reported to be (in part) responsible for the complement activation in AMI [10]–[12]. CRP constitutes a strong cardiovascular risk factor since its plasma levels in both healthy individuals and in patients correlate with occurrence of AMI and are also predictive for the progression of disease once AMI occurs [13]. CRP has been reported to activate the classical pathway of complement by binding C1q [14]. Indeed CRP has been shown to be co-localized to these areas of the heart that are positive for complement deposits [10]. CRP may bind to dead cardiocytes via lyso-phosphatidylcholine that is generated from phosphatidylcholine by phospholipase A_2_ [5]. We have recently shown that cell death by itself carries the potential to activate the complement system, both in the presence or absence of CRP [15]. The complement system has very powerful effects and is therefore kept under control by both membrane bound and soluble complement inhibitors. We have shown previously that one of these fluid-phase complement inhibitors, C4b-binding protein (C4BP) binds to dead cells and provides protection against excessive complement activation [16]–[18]. Also by binding DNA on and released from dead cells it inhibits an inflammatory responses [16]. C4BP is present in serum mostly as a complex with anti-coagulant protein S (C4BP-PS) [19] bound to a beta-chain that together with seven alpha-chains forms one C4BP molecule. Protein S has a high affinity for negatively charged phospholipids and is the module of the C4BP-PS complex that is responsible for most of the binding to apoptotic and necrotic cells. The alpha-chains contain the complement inhibitory capacity and present activated complement components C4b and C3b to factor I for degradation. These alpha-chains also contain the DNA binding site [16].

In the current study we have investigated whether C4BP binds to compromised cardiomyocytes in relation to cardiovascular events. Here we describe that C4BP forms deposits in the heart in several cardiovascular diseases, but based on both the intensity of depositions and the frequency of positivity it is mostly observed in AMI. In AMI lesions it is correlated to areas also positive for C3b. Binding of C4BP to cardiomyocytes peaks, like C3b, between 12 hours and 5 days after the infarction, during the phase in which infiltration of neutrophilic granulocytes in the heart is the most extensive.

## Materials and Methods

### Patients

Samples were obtained from patients who had died following cardiovascular events and were collected in line with the institutional guidelines of the VU Medical Center in Amsterdam, The Netherlands. These patients had given their written informed consent for the use of tissue material for research purposes. The tissue array samples were collected in line with the Lund University ethical comity decision LU-232-01, Lund University, Lund, Sweden, both in accordance with the Helsinki declaration.

Tissue arrays were generated from heart samples patients suffering from AMI, hypertrophy and aorta stenosis. The samples for the tissue arrays were selected based on detailed evaluation of the autopsy reports followed by clinical and histological confirmation. Aorta stenosis, a condition that results in pressure overload induced myocardial hypertrophy, was based on clinical and morphological data (confirmed at autopsy). The hypertrophy group was selected based on increased myocardial mass. This is a heterogeneous group with hypertrophy caused by several underlying conditions including arterial hypertension, ischemic heart disease and left sided valvular leaks with potential overlap between the conditions. We excluded hearts with suspected obstructive hypertrophic cardiomyopathy.

In a separate study tissue samples were stained histochemically allowing correlation between positivity of C4BP and the stage of AMI as well as presence of C3b determined by immunohistochemistry. Three areas were identified in AMI patients: (A) the infarcted area, based on lactate dehydrogenase decolourization, (B) the border zone, right next to A and (C) non affected, remote areas of the same heart.

For quantitative studies samples were grouped according to time after infarction. Group I, n = 9, infarction of 4–12 hours (lactate dehydrogenase decolourization, no influx of neutrophilic granulocytes), Group II, n = 9, infarction of 12 hours–5 days old (acute inflammatory phase), Group III, n = 4, infarction 5–14 days old (chronic inflammatory phase).

### Immunohistochemistry

Paraffin embedded tissue in the form of tissue arrays were deparaffinized using routine procedures and samples were stained for C4BP as described [16]. For quantitative studies in AMI paraffin embedded tissue sections (4 µm thick) were mounted on microscope slides and deparaffinized for 10 minutes in xylene at room temperature and dehydrated through descending concentrations of ethanol. One set of sections was stained with hematoxylin and eosin using routine procedures. Endogenous peroxidase activity was blocked by incubation in 0.3% (v/v) H_2_O_2_ in methanol for 30 minutes. Tissue sections were subjected to antigen retrieval by boiling in 10 mM sodium citrate buffer, pH 6, for 10 minutes in a microwave oven. All antibodies and normal, pre-immune serum were diluted in PBS containing 1% (w/v) bovine serum albumin (BSA). Tissue sections were pre-incubated for 10 minutes with normal swine serum, followed by incubation for 1 hour with C4BP antibodies. The antibodies were raised in rabbits against highly purified human C4BP-PS complex (kind gift from Prof. Dahlbäck, Lund University, Sweden). The antibodies from rabbit serum were first purified using protein A and protein G columns (GE Healthcare) coupled in tandem followed by affinity purification using a column (Affi-gel 10, Biorad) with covalently coupled human C4BP-PS. After washing in PBS tissue sections were incubated for 30 minutes with a biotin-conjugated swine anti-rabbit secondary antibody (DAKO). After washing in PBS slides were incubated with streptavidin–biotin complex (DAKO) for 1 hour. After the incubation with streptavidin–biotin complex C4BP was visualized with 3,3′-diaminobenzidine (0.1 mg/ml, 0.02% H_2_O_2_). Staining for deposition of C3b was performed using mouse mAb C3-15 (kind gift from Prof Hack, The Netherlands) which recognizes the C3d fragment of C3b exactly as described previously [10]. Additional stainings were performed using goat anti-C4 and goat anti-C9 polyclonal antibodies (Quidel) with matched secondary antibodies (DAKO). Slides were counterstained with hematoxylin and mounted with Depex (VWR International).

Samples were analyzed semi-quantitatively in order to discriminate between the several cardiovascular disorders. Samples were graded as: +/−, negative/background positive for C4BP, ++, positive and +++, strongly positive. In addition AMI samples were analyzed quantitatively for the area of positivity/tissue slide by computer-assisted morphometry (QPRODIT).

### Cells, proteins and treatments

Neonatal rat cardiomyoblasts, H_9_C_2_ cells (ATCC) were cultured as before [20]. Briefly, cells were grown at 37°C in 5% CO_2_ in Dulbecco's modified Eagle's medium supplemented with 10% (vol/vol) heat-inactivated fetal calf serum, 100 IE/ml sodium penicilline, 100 mg/L streptomycin, and 2 mM L-glutamine, all from Invitrogen. For experiments cells grown to a confluency of 60–80% were used. Cells were detached from the culture flasks using versene (Invitrogen). Cells were either analyzed as live cells or were induced into apoptosis by incubating with staurosporine at 0.5 µM for 15 hours at 37°C or induced into necrosis by incubating the cells at 56°C for 30 minutes in RPMI without FCS. Cell death was confirmed by flow cytometry using Annexin V and Via-Probe staining (BD Biosciences). Alternatively, H_9_C_2_ cells were exposed to 3 hours of acute hypoxia (0.1% oxygen) followed by 1 hour of reoxygenation. To achieve hypoxic conditions, cells were kept in a humidified hypoxia chamber, the Hypoxia Workstation 400 (Ruskinn Technology), connected to a Ruskinn gas mixer module supplying 95.9% N_2_, 5% CO_2_ and 0.1% O_2_. Normoxic or hypoxic cells were incubated with C4BP-FITC or prothrombin-FITC both at 0.1 mg/ml and binding was analyzed by flow-cytometry. The C4BP-PS complex [21] and prothrombin [16] were purified from normal human plasma and labeled with FITC as described before. The mean fluorescence intensity (MFI) of C4BP-FITC was normalized to the MFI of prothrombin-FITC, used here as a negative control and the ratios for independent experiment (n = 4) were averaged and expressed as means +/− standard deviation (SD).

### Flow cytometry

Binding of C4BP to cardiomyocytes was analyzed by flow cytometry. Following washing cells were incubated with increasing concentrations of purified human C4BP-PS (0–25 µg/ml) diluted in binding buffer (10 mM HEPES, 150 mM NaCl, 5 mM KCl, 1 mM MgCl_2_, 1.8 mM CaCl_2_), for 30 minutes at room temperature. Following washing with binding buffer cells were incubated with a rabbit anti-human C4BP antibody, followed after washing with swine anti-rabbit FITC labeled antibodies (DAKO). The cells were analyzed using FACS Calibur (BD Biosciences).

### Statistical analysis

Data analysis was performed with GraphPad Prism software. The data were normally distributed and Student's t-test was used to calculate the significance of the differences. A p value (two tailed) of less than 0.05 was considered to be significant.

## Results

### C4BP deposition occurs in several cardiovascular diseases but is strongest in AMI

**Figure 1:**
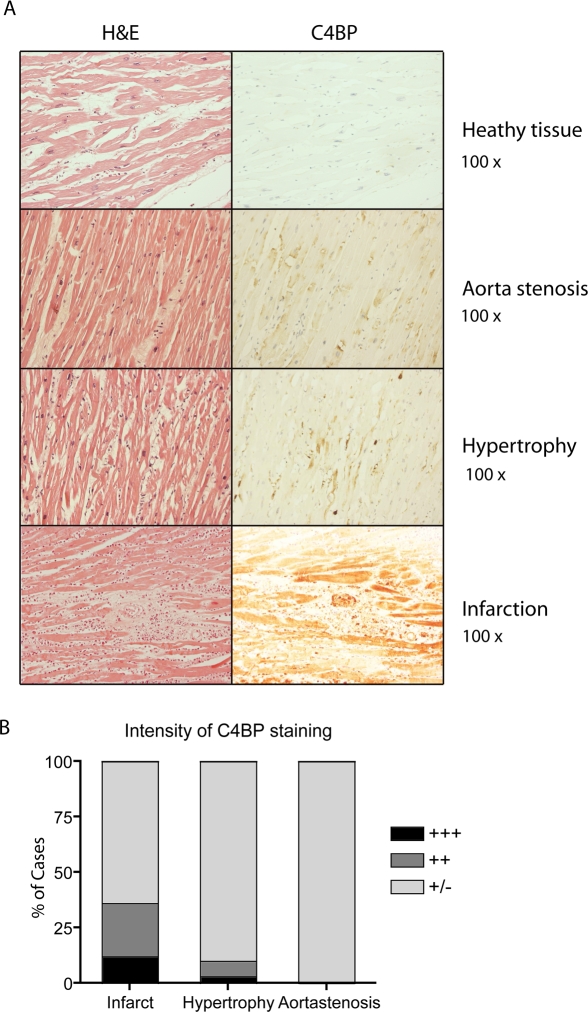
C4BP is present on compromised heart tissue. (A) Tissue arrays of heart tissue from patients suffering from aorta stenosis, hypertrophy and AMI were stained with hematoxylin and eosin or for C4BP. Representative examples are shown. Original magnification 100×. (B) Semiquantitative analysis of the percent of cases subgrouped for the intensity of C4BP staining.

By using tissue arrays of several cardiovascular diseases we set out to determine if C4BP deposition occurred in cardiac disease. We have compared samples from patients diagnosed with cardiac hypertrophy, aorta stenosis and AMI. The healthy parts of patient heart tissue showed only very modest C4BP positivity, which appeared not to be cell associated and may reflect C4BP present in blood (Figure 1). C4BP immunostaining was relatively weak in aorta stenosis and hypertrophy with some staining on individual cardiomyocytes and of the space between the cells (Figure 1). In contrast, in AMI a strong staining of C4BP was observed on all cardiocytes in the entire region. By semi quantitative scoring of the intensity of staining of those tissue arrays we observed that both the frequency and the positivity for C4BP were highest in AMI compared to hypertrophy and aorta stenosis (Figure 1B). Based on these results we focused further experiments on AMI.

### C4BP is deposited on affected cardiomyocytes

**Figure 2:**
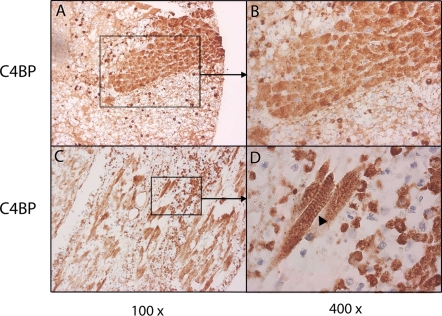
C4BP binds to individual cardiomyocytes. Compromised cardiomyocytes stain positive for C4BP shown in both cross sectional (A,B) and sagital view (C, D). Boxed areas in A and C (original magnification 100×) are shown at higher magnification in B and D (original magnification 400×). Arrowhead in D shows cross-striation, typical for cardiomyocytes.

**Figure 3:**
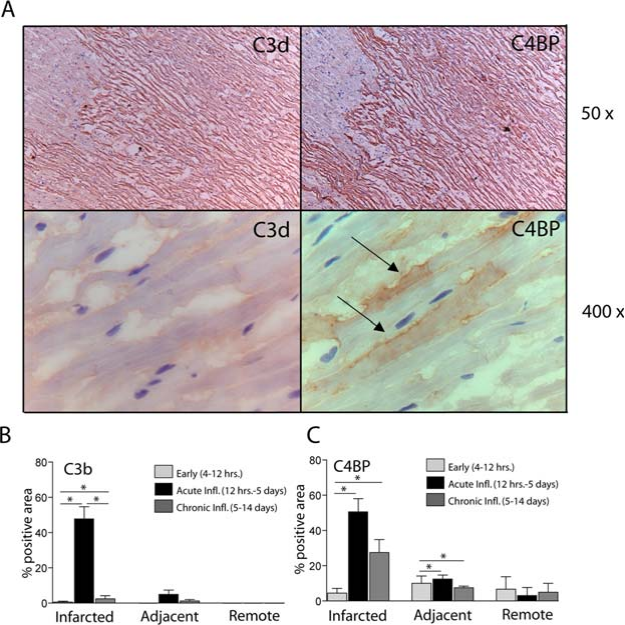
Binding of C4BP is strongest during the acute inflammatory phase of AMI and correlates to C3b. (A) Consecutive sections were stained for C4BP and C3d and display binding to the same area of compromised heart tissue (original magnification 50×). Higher magnification pictures of the same area show individual cardiomyocytes positive for both C3d and C4BP (original magnification 400×). Arrows indicate positive cardiomyocytes. (B) Quantification of the % of positive area for C4BP in the infracted area, the adjacent area or remote areas during the early, acute inflammatory or chronic inflammatory phase of AMI. (C) Quantification of the % positive area for C3b on the same locations and time points.

In AMI heart tissue C4BP localization was limited mostly to jeopardized cardiomyocytes (Figure 2). Depending on the angle of cutting, the sections displayed a banded pattern on the cardiomyocyte (Figure 2C, D). The positive staining of the myofiber elements included the sarcolemma, cytoplasm and cross-striation. While in some cases there was a gradual change in intensity of the C4BP positivity (Figure 1A), in other cases there was a sharp boundary between positive and negative areas (Figure 2A). In fact, at a higher magnification it is possible to see adjacent cells of which one is positive and the other is negative for C4BP (Figure 3A), indicating that the cell needs to go over a certain threshold of changes before it binds C4BP.

### C4BP positivity extends beyond positivity for C3b

To test if the localization of C4BP is related to the affected area, samples were stained for the presence of C3b and analyzed by computer assisted scoring of area of positivity. Based on several morphological criteria as well as patient records it was possible to estimate the time after occlusion and to relate the findings of C4BP and C3d intensity to the time after infarction. The positivity for C4BP and C3b was scored in the infarcted area, the area just adjacent to this ( = border zone area) and in remote areas. Indeed positivity for C4BP was mainly observed in the areas with extensive cell death, but it was also found on locations in the border zone and even the non-affected tissue (Figure 3A, B). Staining of consecutive sections for C3b revealed that C4BP was located to similar areas as C3b except for the areas in the non-affected tissue.

Both C4BP and C3b were not present in samples of very early infarction samples but both were deposited during the acute inflammatory phase (12 hours to 5 days old). C3b positivity diminishes after this period, while C4BP stays positive over a more prolonged period of time. The fact that C4BP was observed in the adjacent tissue while C3b was not deposited there, could indicate that C4BP present on this location is sufficient to protect against complement activation and deposition of C3b on the adjacent tissue.

The pattern of staining of microscopical infarction areas for C3b, were identical to IgM and CRP [22]. In these larger infarction areas C4BP had the same staining pattern as C3b, but interestingly also stained individual cardiomyocytes, that were morphologically normal beyond that microscopical area (Figure 3B, C).

Sections were also stained for C4 and C9, as a marker for deposition of the membrane attack complex, and we observed that deposition of C4 and C9 peaks during the acute inflammatory phase, just as is the case for C3b and C4BP (data not shown).

**Figure 4:**
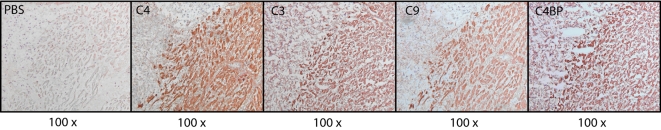
C4, C3b and C9 are also present on the affected tissue. Representative pictures of consecutive sections of heart tissue of an AMI sample stained for C4, C3b, C9 and C4BP or with secondary antibody only (PBS).

By staining consecutive sections for the presence of C4, C3b, C9 and C4BP we observed that both C4 and C9 are present on the affected tissue in a similar pattern as C3b (figure 4A). Collectively these data indicate that complement is activated on the affected heart tissue, including deposition of C4 and C3b up to the formation of the membrane attack complex and that C4BP, which is deposited strongly in the affected and weakly in the close unaffected area, could function as a protective factor by inhibiting excessive complement activation.

### Cultured cardiomyocytes bind C4BP mainly following cell death

**Figure 5:**
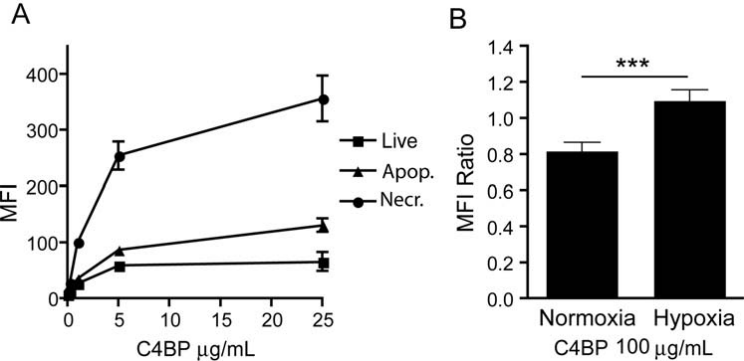
C4BP binds strongly to dead but not live cardiomyocytes. (A) Rat cardiomyocyte cell line H_9_C_2_ was used to study the binding of human C4BP to live, apoptotic or necrotic cardiomyocytes. Binding of C4BP to these cells was analyzed by FACS and is displayed as mean fluorescence intensity (MFI). Increasing concentrations of C4BP lead to increased binding to apoptotic and especially necrotic cardiomyocytes with no binding to live cells. (B) Slightly enhanced binding of C4BP to hypoxic H_9_C_2_ cells compared to normoxic. The MFI of C4BP-FITC was normalized to the MFI of prothrombin-FITC, used as a negative control and the ratios for independent experiment (n = 4) were averaged and expressed as means +/− SD.

As a model for human cardiocytes we have used rat cardiomyocyte cell line H_9_C_2_. These cells were cultured *in vitro*, harvested and rendered apoptotic by addition of staurosporine, or necrotic by incubating in heat (30 minutes, 56°C). Live, late-apoptotic and necrotic cells (as determined by staining with annexin V and Via-probe) were incubated with increasing concentrations of purified C4BP-PS and the binding was analyzed by flow cytometry. C4BP-PS displayed a background binding to live cardiomyocytes but binding of C4BP-PS was increased in a dose-dependent manner on apoptotic and especially necrotic cells (Figure 5A). These data indicate that cell-death is one of the mechanisms by which C4BP binds to the heart tissue. In addition to cell death we have also analyzed the binding of C4BP to H_9_C_2_ cells cultured in hypoxic conditions. The binding of C4BP to normoxic H_9_C_2_ cells is low and increased only marginally on the hypoxic cells (Figure 5B). Under these conditions of hypoxia we did not observe apoptotic or necrotic H_9_C_2_ cells as determined by staining for annexin V and Via-probe (not shown).

## Discussion

C4BP, the main fluid-phase inhibitor of the classical and lectin pathway of complement activation is present in affected tissue of patients suffering from AMI. C4BP was shown previously to bind to dead cells and provide protection against excessive complement activation and inflammation otherwise evoked by these cells [15]–[18]. C4BP as a complement inhibitor limits complement activation but does not provide a complete block [19]. Indeed this is what we also observed in the current study, C4BP co-localizes with C4, C3b and C9 in the damaged heart tissue, which indicates that in the presence of C4BP there is still complement activation, but it would have been much more extensive if C4BP was lacking as we have shown previously for other cell types [16]. The observation that C4BP is found on the surrounding tissue in the absence of C3b may indicate that the levels of complement inhibition by C4BP are sufficient there to protect against complement activation but not in the larger infarct areas. To firmly establish the relative importance of C4BP in these lesions one would have to use animal models of AMI, using C4BP deficient animals.

Although the complement system contributes to the tissue damage it is not easy to discriminate this from the beneficial effects of complement in terms of waste disposal. Nonetheless, experiments using complement deficient animals and complement inhibiting drugs revealed reduced infarction size in the absence of complement activation indicating that complement does contribute to pathology (reviewed by [9]). Complement activation can have several effects on the infarcted heart, e.g. lysis of cells by means of the membrane attack complex, release of C5a that attracts neutrophils but it can also influence the function of the myocardium itself. Several studies have found activated complement components on myocardium especially after sufficient time of infarct duration of more than 12 hours [10], [23].

Both C4BP and factor H have been reported to play a role in cardiovascular disease such as atherosclerosis. C4BP is present and inhibits classical pathway activity in atherosclerotic lesions [24] and in the necrotic areas of advanced atherosclerosis [16]. Factor H has also been located in atherosclerotic lesions and has been implicated as a protective factor [25]. In the atherosclerotic plaques synthesis of complement factors is upregulated but not that of complement inhibitors [26] indicating that most likely the inhibitors are localized there from blood.

Both C3b and CRP have been observed in the same binding pattern as C4BP, with a cytoplasmic staining and cross-striation [10]. This may indicate that these proteins are in fact located both on the outside and on the inside of these cells. For CRP it was reported that it does not stain nuclear elements and it seems that also C4BP staining does not reveal a staining for nuclear elements, which is interesting since both CRP and C4BP are reported to bind DNA. The binding of C4BP to dead cells in the heart tissue may be easily explained by the binding of the C4BP-PS complex to phosphatidylserine, as we reported before for other cell types [16]–[18]. However, the binding of C4BP to seemingly healthy tissue surrounding the dead area is less clear, either these cells have undergone a certain extend of membrane reorganization or flip-flop that allows the binding of the C4BP-PS complex. Another possibility would be that hypoxic cells show increased binding of C4BP but we only observed a small increase in C4BP binding at high C4BP concentrations. However, it is clear that C4BP binding in these areas is not dependent on its ligands complement C4b and C3b since they are absent from these areas.

To our knowledge C4BP has not been used to treat or prevent pathology in the context of cardiovascular disease, whereas supra-normal levels of C4BP may provide a relatively safe approach. Clearly more studies are needed before such experimentation can be translated into a clinically useful approach.

In conclusion: in this study we provide evidence that fluid-phase complement inhibitor C4BP binds to compromised heart tissue. The deposition is strongest in AMI compared to aorta stenosis and ischemic heart disease and it takes place during the inflammatory phase of AMI. C4BP positivity extends beyond the area positive for C3b, which suggests that C4BP is a functional complement inhibitor in AMI and limits complement activation to the most affected tissue.
